# Supplementary material for: Patterns of Intron Gain and Loss in Fungi
Source: PLoS Biol. 2004 Nov 30;2(12):e422. doi: 10.1371/journal.pbio.0020422 (PMC532390; doi:10.1371/journal.pbio.0020422)
Supplement: Table S1 — Also available at http://genes.mit.edu/NielsenEtAl/. (4.3 MB ZIP). [file pbio.0020422.st001.zip › NielsenEtAl/html/453.html]

AN4717.1.NCU00682.1.MG02832.1.FG08729.1


```
 CLUSTAL W (1.82) Multiple Sequence Alignments - Introns Inserted


Sequence 1: NCU00682.1	415 aa
Sequence 2: FG08729.1	392 aa
Sequence 3: MG02832.1	408 aa
Sequence 4: AN4717.1	396 aa
Alignment Length: 425 aa
Number Identitical Residues: 175 aa
Alignment Score (without introns) 8652


MG02832.1 	MAAALAHSSG-----MIPSMVGVPPSTSYENGSGGNSS-------TTAGHGSVNDGSRTS
NCU00682.1	MAATVASSHPFGGGLHPLQAKGLLAAAQQRQQQQQTQSQPQLHPCMLSQHHSYQNDQRRN
FG08729.1 	MAATVAPSHAAG----LPSSSGIPASS-----------------------HSVNSHSHSQ
AN4717.1  	MASGTVGPSP------PSGVQGSSASDNSGNPTPGEIHDR-----EKRTVASVRPQTQRH
          	**:  . .             *  .: .  .       .            * .   :  

MG02832.1 	RNALQGDLKSDAASAQSQNRKLQLSDFKKVRTLGTG1TFARVCLVRPSNPQNE-TERNK-
NCU00682.1	DPSQTQKNNDTSGGFEMATRKPRLSDFHRIRTLGTG1TFARVVLVRPANGTEI--DRQK-
FG08729.1 	DVSGASTSSAPSP--LPQTRKFNLRDFRRVRTLGTG1TFARVCLVRPAATAHIPLDHQLN
AN4717.1  	PNPFVITERPEEKQLGISTRQLSVRDFVLLKTLGTG1TFARVWLARLRDDKTR---PEK-
          	  .               .*:  : **  ::***** ***** *.*           :  

MG02832.1 	--VFALKILRKSEV~VKLKQIDHVRHERAILADVSGFPFITNMLASFSDHDFLYIV~LDY
NCU00682.1	--VYALKILRKTEV1IRLKQIDHVRHERQILQDVTGHPFITSLQASFSDHDFLYLL~LDY
FG08729.1 	PEVYALKILRKPEV~IRLKQVDHVRHERAILADVAGHPFITTLIASFSDQDSLYML~LDY
AN4717.1  	--VYALKILRKADV1IKLKQVEHVRNERKTLADVSGHPFITTLIASFSDSQSLYML0LDY
          	  *:*******.:* ::***::***:**  * **:*.****.: ***** : **:: ***

MG02832.1 	VPGGELFSYLRKYRRFDEDMARFYAAEIVLVLEYLHEAQDGVAYRDLKPENLLLDGQGHI
NCU00682.1	IPGGELFTYLRKYRRFDEEMARFYAAEIVLVLEYLHEEQGGIAYRDMKPENLLLDADGHI
FG08729.1 	VPGGELFTYLRKLRRFEEPVARFYAAEIVLVLEYLHEQQGGIAYRDLKPENLLLDQDGHI
AN4717.1  	CPGGEIFSYLRRARRFNENTSKFYAAEITMTIEFLHDAEG-VVYRDLKPENILLDADGHI
          	 ****:*:***: ***:*  ::******.:.:*:**: :. :.***:****:*** :***

MG02832.1 	KLVDFGFAKRLGGRRDGDNSGTQETYTLCGTPEYLAPEVIHNKGHTTAVDWWALGILIYE
NCU00682.1	KLVDFGFAKRLGYN---DVERPVETYTLCGTPEYLAPEVIQNKGHTTAVDWWALGILIYE
FG08729.1 	KLVDFGFAKRLGYR---EDNRPAETYTLCGTPEYLAPEVIQNKGHTGAVDWWALGILIYE
AN4717.1  	KLVDFGFAKQIGDR---------ETYTLCGTPEYLAPEVIHNSGHGLAVDWWALGILIYE
          	*********::* .         *****************:*.**  *************

MG02832.1 	FLTGYPP~FWHQNPIEIYKQ2IVEKPVVFPQDPPISPNAQDIIRQFCTVDRSRRLGNISG
NCU00682.1	FLTGYPP2-RKSTVTKTQVR~IVEKPVLFPSSTEISEEAKDIIRSFCTVDRTMRLGNMSG
FG08729.1 	FLTGYPP~FWHQNPIEIYKQ2ILEKPVVFPQDPSLSEEAKDIIRSLCTVDRSRRLGNISG
AN4717.1  	FLVGQPP~FWDQNPMRIYEQ~IVEGHIRYPSN--MSPAAQNIISLLCKTNPAERLGHISG
          	**.* **   ...  .   : *:*  : :*..  :*  *::**  :*..: : ***::**

MG02832.1 	GAARVKEHPFFEGVDWEAIYSRKFPGPILPPIRYPGDAQCFDIYPEED-VGKDPYTDEMA
NCU00682.1	GAARVKAHPWFKGVDWEAVEQRRHKGPIIPHLSHPGDASCFDVYPEQD-VHNEAYTEEMF
FG08729.1 	GAARVKSHPFFQDTDWDEILDRRHKGPIQPPIRYPGDAQCFDVYPEDD-GTREPYGREMA
AN4717.1  	GSARVRTHPFFENINWDDLFYRRIKGPIIPRVDHPADTGNFEEYPDPDPKSQTIYTEDMR
          	*:***: **:*:. :*: :  *:  *** * : :*.*:  *: **: *.  .  *  :* 

MG02832.1 	QKYDHYFQDF
NCU00682.1	EKYEKYFGDF
FG08729.1 	EKYDPYFADF
AN4717.1  	SKYETLFSDF
          	.**:  * **
```
